# Enhanced Th1/Th17 Functions of CD161^+^ CD8^+^ T Cells in Mucosal Tissues of Rhesus Macaques

**DOI:** 10.1371/journal.pone.0157407

**Published:** 2016-06-16

**Authors:** Namita Rout

**Affiliations:** Tulane National Primate Research Center, Tulane University, Covington, Louisiana, United States of America; University of Pittsburgh Center for Vaccine Research, UNITED STATES

## Abstract

Expression of the C-type lectin-like receptor CD161 by human T cells is associated with type-17 responses, which play critical regulatory roles in immunity and inflammation at mucosal sites. However, the functions of CD161-expressing T cells in macaques, the pre-clinical model of several human diseases, remain unknown. This study examined the phenotypic and functional characteristics of CD161^+^ T cells in peripheral blood, mucosal tissues and lymph nodes of rhesus macaques. Majority of CD161-expressing T cells in peripheral blood and lung/intestinal mucosal tissues of rhesus macaques were found to be CD8^+^CD4^–^ in phenotype. There was a significant enrichment of CD161^+^CD8^+^ T cells in the lungs and colonic mucosa (16.1%±6.6 and 16.8%±5.7) in comparison to peripheral blood (4.2%±1.2) and mesenteric lymph nodes (1.3%±0.8). Regardless of the tissue compartment, CD161^+^CD8^+^ T cells mainly comprised of γδ T cells and TCR Vα7.2^+^ MAIT cells (up to 80%), and displayed Th1 and Th17 cytokine responses to mitogen stimulation. Mucosal CD161^+^CD8^+^ T cells were characterized by very high expression of CD69, a recent activation marker that is preferentially expressed on tissue resident cells. Furthermore, lung and colonic mucosal CD161^+^CD8^+^ T cells showed enhanced IFN-γ, IL-17, and Perforin production in comparison to those in blood. Thus, macaque CD161^+^CD8^+^ T cells represent mucosal tissue-homing innate-like CD8^+^ T-cell populations with Th1/Th17 type cytokine and cytotoxic effector functions that can potentially enhance the recruitment of adaptive immune cells and control initial pathogen burden/dissemination in tissues. Analysis of their role in early immune responses to mucosal pathogens will be valuable in the design of vaccines and therapeutics.

## Introduction

CD161 is a C-type lectin-like receptor that belongs to the Killer cell lectin-like receptor subfamily (KLRB1), also known as NKR-P1. Originally identified as the surface NK1.1 antigen in rodent natural killer (NK) cells [1], it was later found to be expressed in human NK cells [2]. CD161 is expressed by a broad range of lymphocytes, including NK cells, CD4+, CD8+, γδ+ T-cells, NKT cells, and mucosal-associated invariant T (MAIT) cells [2,3]. It acts both as an inhibitory receptor and as a co-stimulatory molecule for proliferation, cytolytic activity and IFN-γ production by NK cells and T cells [2,4,5]. Recent studies have determined the significance of CD161 expression in the type-17 effector functions of human T cells and regulation of tissue-specific immune responses [3,6–8]. CD161 is considered as a marker of human Th17 cells that secrete both IL-17A and IL-17F and express the transcription factor retinoic acid-related orphan receptor (ROR)-γt [9,10]. Likewise, CD8^+^ T cells expressing high levels of CD161 secrete IL-17 and these IL-17-secreting CD161^high^CD8^+^ T cells (Tc17 cells) are polarized toward the type-17 lineage [6]. However, the biological functions of the receptor in T cells including its roles in inflammation and infections remain to be fully defined.

CD161 expression is a common feature of innate T cell subsets including invariant natural killer T (iNKT) cells, γδ T cells, and MAIT cells, at levels higher than conventional CD4^+^ and CD8^+^ T cells [11]. Indeed, a very high level of CD161 expression on human CD8^+^ T cells is considered as a surrogate marker for MAIT cells [12], an immune subset analogous to CD4^+^ Th17 cells [6]. These CD8^+^CD161^++^ T cells express a pattern of molecules associated with Th17 phenotype, including expression of RORγt, CCR6, and IL-18R along with cytokines IL-17, IL-22, and IFN-γ [6]. However, in the presence of IL-12, human Th17 cells can produce IFN-γ and IL-17 along with the upregulation of the Th1 transcription factor T-bet and downregulation of the Th17 transcription factor RORγt, thus demonstrating that these subsets may share a developmental relationship with a degree of plasticity that allows them to carry out distinct functions in different cytokine milieu [13]. Even though, CD161 expressing T cells vary in phenotype, functions and recognition of antigens and antigen presenting molecules, these cell subsets share a common transcriptional signature and display innate-like function independent of TCR-stimulation [7]. Additionally, human CD161-expressing CD8^+^ antiviral memory T cells exhibit polyfunctional and gut tissue-homing properties with enhanced effector functions, including cytokine production and high levels of cytotoxic mediators along with the expression of transcription factors T-bet and eomesodermin (EOMES), suggesting their eminent role in antiviral immunity and vaccine responses [14].

Since CD161 represents the single human ortholog of the family of NKR-P1 genes in rodents [2], with only 46–47% homology with the mouse NKR-P1 proteins, study of CD161-expressing T cells is currently restricted only to primates. Although tissue-resident CD161^+^ T cells have been reported in human liver and intestine [8,15,16] and have been implicated in local immunity, the phenotypic characteristics and immunological functions of these cells are not known in nonhuman primates. Characterization of CD161^+^ T cell functions in nonhuman primates would be valuable to address their role in early immune responses to viral and bacterial pathogens in tissue sites that are challenging to access in human disease. Therefore, this study investigated the phenotype and function of CD161-expressing T cells in peripheral blood, lymph nodes, and mucosal tissue compartments of the nonhuman primate model of rhesus macaques. The data suggest that macaque CD161^+^ T cells are functionally analogous to human CD161^+^ T cells displaying Th1/Th17 type cytokine responses and are enriched in mucosal tissues including lung and colon.

## Materials and Methods

### Ethics statement

This study was carried out in strict accordance with the recommendations by the Guide for the Care and Use of Laboratory Animals of the National Institutes of Health. The animal protocol was approved by the Tulane University Animal Care and Use Committee. All the animals in this study were housed at the Tulane National Primate Research Center (TNPRC) and under the care of TNPRC veterinarians in accordance with the standards incorporated in the Guide to the Care and Use of Laboratory Animals (NIH) 78–23 (Revised, 1996).

### Animals and tissue sampling

Rhesus macaques utilized for obtaining blood and tissue samples in this study were housed at the TNPRC. Heparinized blood samples were obtained from healthy Indian rhesus macaques. Tissue specimens were obtained from uninfected rhesus macaques undergoing euthanasia. Lung tissue, colon segment and blood were collected from all animals at the time of necropsy.

### Isolation of lymphocytes

Blood collected in heparin vacutainer tubes (Becton Dickinson Vacutainer systems, Franklin Lakes, NJ) was processed immediately. Peripheral blood mononuclear cells (PBMC) were separated by density gradient centrifugation (Lymphocyte Separation Medium; MP Biomedicals Inc., Solon, OH) at 1500 rpm for 45 minutes and used for phenotyping and *in vitro* functional assays. For the isolation of lymphocytes from colon tissue, one-inch segment was collected in 50 ml tubes with ice-cold RPMI (Fisher Scientific) and immediately transported to the laboratory. Fat and blood vessels were trimmed. Mucus and gross debris were removed and the specimens were further washed twice in cold PBS (pH 7.4). Tissue samples were cut into small pieces (approximately 0.5–1 cm^2^) and processed as previously described [17]. In brief, tissues were treated with EDTA with shaking at 37°C, followed by digestion in complete RPMI medium containing 5% fetal calf serum (FCS) (RPMI-5) containing 75 units/ml of Type II collagenase (Sigma-Aldrich) with shaking at 37°C to release cells from lamina propria. For enrichment of lymphocytes, lamina propria cells were centrifuged over discontinuous Percoll (Sigma-Aldrich) density gradients followed by washing with PBS. All isolated cells were washed and re-suspended in complete RPMI-10 medium containing 10% FCS before staining or stimulation for functional assays. All cells were >90% viable by trypan blue dye exclusion method. Lymphocytes from mesenteric lymph nodes and lung tissue were isolated by cutting into small pieces (approximately 0.5–1 cm^2^) in a petri-dish and treating by mechanical ways (squeezing with the back of a 10 mL syringe plunger) in cold PBS, and then the suspension was filtered through 75 μm filters to remove the major tissue fragments, and get the lymphocytes with > 70% purity.

### Flow cytometry

Multi-color flowcytometric analysis was performed on cells according to standard procedures using anti-human mAbs that cross-react with rhesus macaques. For immunophenotyping analysis PBMC were stained with the antibodies CD45 BV510 (BD clone D058-1283), CD3 APC-Cy7 (BD clone SP34 clone), CD4 QDot 605 and CD8 QDot 655 (NHP Reagent resource), CD161 PE-Cy7 (eBioscience clone HP-3G10), TCR γδ FITC (BD clone B1), TCR Vα7.2 BV421 (Biolegend clone 3C10), PBS-57 loaded CD1dTM APC (NHP Reagent resource), CD28 ECD (28.2 clone; Immunotech), and CD95 APC (BD clone DX2). For analysis of trafficking marker and cytokine receptor expression, the antibodies CCR5 APC (BD clone 3A9), CCR6 PerCP-Cy5.5 (BD clone 11A9), CXCR3 Alexa 700 (BD clone 1C6/CXCR3), CXCR5 Alexa 488 (BD clone RF8B_2_), IL-18Rα PE (R&D clone #70625), CD69 ECD (Coulter clone TP1.55.3) and α_4_β_7_ APC (NHP Reagent resource) were used. For evaluation of cytokine production, mononuclear cells from blood and tissues were stained extracellularly with CD3/CD161/CD8/CD4/γδTCR/Vα7.2TCR followed by a permeabilization and intracellular cytokine staining with CD69 ECD, IFN-γ PE-Cy7 (BD clone B27), IL-17 PerCP-Cy5.5 (eBioscience clone eBio64DEC17) and TNF-α Alexa 700 (BD clone MAb11) were utilized. Data was acquired on a BD Fortessa with FACSDiva software.

Analysis of the acquired data was performed using FlowJo software (version 9.6.2; TreeStar, Ashland, OR). For experiments measuring polyfunctional responses, Boolean gating was used to partition cells into specific response categories followed by data analysis using PESTLE v1.7 and SPICE 5.3 software (provided by NIAID/ NIH in collaboration with Dr. Mario Roederer).

### Immunofluorescent staining of cells

Surface staining was carried out by standard procedures. Briefly, 1 to 2 million cells resuspended in 100 μl stain media (PBS with 2% FBS) were incubated with surface antibodies for 30 min at 4°C. After washing with stain media, the cells were fixed in 2% paraformaldehyde. All intracellular cytokine staining (ICS) assays were carried out on cells that were stimulated overnight. Following 16 h incubation, cells were washed with PBS and incubated for 15 minutes at 37°C in 0.5 mM EDTA/PBS, stained for surface markers in wash buffer for 30 min at 4°C, washed and then fixed and permeabilized using the Invitrogen Fix/Perm reagents (CALTAG). Permeabilized cells were stained intracellularly with the requisite antibodies. Cells were then washed in wash buffer and fixed in 2% paraformaldehyde (PFA).

### Functional Analyses

For ICS, 1 million PBMC and tissue lymphocytes were stimulated with Phorbol-12-Myristate-13-Acetate (PMA, 10 ng/ml, Sigma-Aldrich) and Ionomycin (1 μg/ml, Sigma-Aldrich) in the presence of brefeldin A (5 μg/ml, Sigma-Aldrich) for 12–16 h at 37°C in 5% CO_2_. After activation, the cells were washed and stained appropriately as earlier described [18].

For analysis of secreted cytokines, cells were incubated with medium alone, or in the presence of either 100ng/ml SEB (Sigma-Aldrich), or 50 ng/ml phosphoantigen (E)-1-hydroxy-2-methylbut-2-enyl 4-diphosphate (HMBPP, Sigma-Aldrich), or PFA-fixed *E*.*coli* (DH5α) to represent unstimulated cells, polyclonal stimulation of superantigen-specific T cells, specific stimulation of Vδ2 γδ T cells, and MAIT cells respectively. Prior to the experiment aliquots of 1×10^9^ E.coli (DH5α)/ml were fixed for 5 min in 1% PFA and stored at −80°C. After thawing and washing, *E*. *coli* was added in a bacteria/cell ratio of 100∶1 as described elsewhere [19]. 24 h culture supernatants were collected from lymphocytes stimulated at 20 × 10^6^ cells/ml; 100 μl/well in 96-well cell culture plates and stored frozen at −20°C. TNF-α, IFN–γ, IL-13, and IL-17 were detected in supernatants by using ELISA for monkey cytokines (U-CyTech, Utrecht, Netherlands), performed according to the manufacturer’s instructions. Levels of cytokines (pg/mL) were interpolated from standard curves. Data are expressed as mean ± SEM cytokine production by lymphocytes from each tissue type.

Alternatively, *ex vivo* analysis of secreted Perforin and IFN–γ was carried out by ELISPOT assay (Mabtech) in lymphocytes from blood and tissues that were directly stimulated with SEB, HMBPP and fixed *E*.*coli* in ELISpot wells at an input of 2 × 10^5^ cells/well as previously described [20]. The capture- and biotinylated detector-matched mAb pair for IFN-γ were clones MT126L and 7-B6-1, respectively and for Perforin were clones Pf-80/164 and Pf-344, respectively. Briefly, unfractionated lymphocytes were plated on anti–IFN-γ/Perforin–coated sterile 96-well polyvinylidene difluoride MultiScreen-IP plates (Millipore, Bedford, MA) and stimulated overnight with antigens. Spots were developed by successive incubation with streptavidin-alkaline phosphatase followed by the substrate NBT/5-bromo-4-chloro-3-indolylphosphate buffer (Moss, Pasadena, MD). Spots were counted on a KS ELISPOT Automated Reader System (Carl Zeiss, Thornwood, NY) using KS ELISPOT 4.2 software (performed by ZellNet Consulting, Fort Lee, NJ). Frequencies of responding cells obtained after subtracting background spots in negative control wells were expressed as spot-forming cells (SFC) per million PBMC. ELISPOT responses to individual stimulating antigens that were >2-fold above those of negative control wells, and >50 SFC/10^6^ cells were considered positive.

### Statistical analyses

Statistical differences between tissue compartments within the same group of animals represented in bar graphs were determined by use of the Wilcoxon matched-pairs signed rank test. *P*<0.05 was considered statistically significant. All statistical analyses were performed using the GraphPad Prism software version 6.0b (GraphPad Software, Inc., La Jolla, CA). All data are presented as mean ± SEM.

## Results

### CD161^+^CD8^+^ T cells comprise a major population of circulating CD161^+^ T cells in rhesus macaques

**Fig 1:**
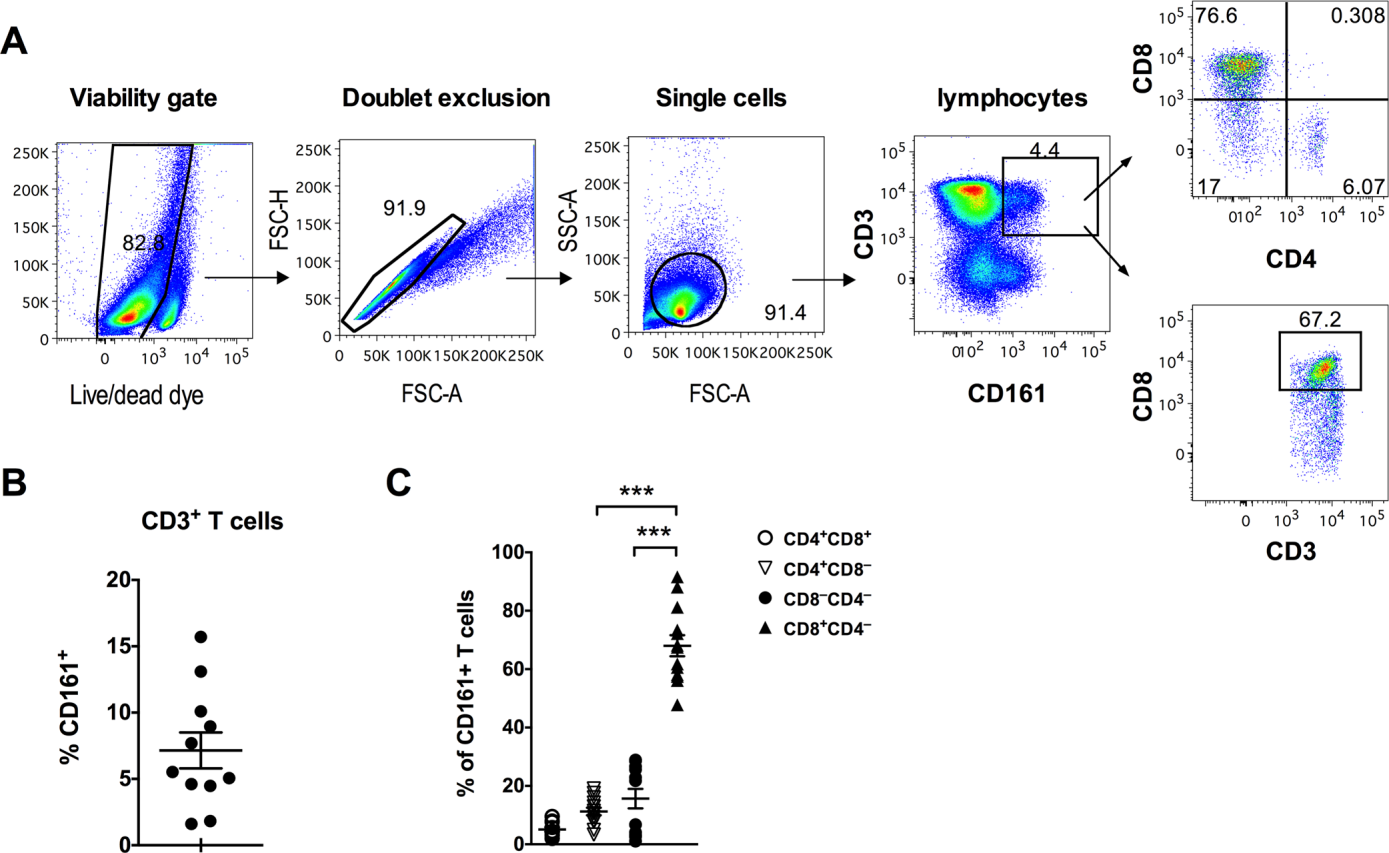
Frequency and CD4/CD8 co-receptor expression of peripheral blood CD161^+^ T cells in rhesus macaques. (A) Representative flow plots showing gating strategy used for detection of CD161^+^CD8^+^ T cells. (B) CD161^+^ T cell frequencies in PBMC of healthy rhesus macaques (n = 11). (C) Subset distribution of circulating CD161^+^ T cells based on the expression of CD4 and CD8 co-receptors in healthy rhesus macaques (n = 11). Asterisks denote significant differences calculated by Wilcoxon matched-pairs signed rank test (*** p<0.001).

Previous studies have identified three subpopulations of CD3^+^CD161^+^ T cells within the peripheral blood of humans: CD161^++^, CD161^+^, and CD161^low^ [7]. In this study, anti-CD3/CD8/CD4 and anti-CD161 monoclonal antibodies were used to evaluate the frequency and sub-populations of CD3^+^CD161^+^ T cells in peripheral blood of healthy rhesus macaques. The gating strategy is shown in Fig 1A. Unlike human CD3^+^CD161^+^ T cells that demonstrate variable expression levels of CD161 (low/intermediate/hi) dividing them into distinct subpopulations [6,11,21], there was a lack of clear distinction in CD161 expression on the circulating T cells of rhesus macaques (representative flow cytometric plot in Fig 1A). However, similar to that reported in human CD161-expressing T cells, there was a trend of increase in CD161 median fluorescence intensity (MFI) from total T cells<γδ T cells<iNKT cells<MAIT cells (S1 Fig).

CD161^+^ T cells comprised 7.15% (range: 1.6–15.7%) of the total circulating CD3+ T cell population (Fig 1B). The circulating CD161^+^ T cells displayed a predominant memory phenotype (mean = 98% CD95^+^) compared to 90% of CD95^+^ memory cells in total T cells in the same group of individuals (S2 Fig). Majority of the CD3^+^CD161^+^ T cells were CD8^+^ (mean = 68%, range: 48–92%), followed by 15.7% (range: 1–28.6%) of CD4^–^CD8^–^ T cells and only 11.2% (range: 3.3–19.1%) of CD4^+^ T cells (Fig 1C). Overall, similar to that reported in human blood T cells [7], CD161 expression was mainly found on memory CD8^+^ T cells in circulating blood of adult healthy rhesus macaques. Thus, further analyses of the CD161^+^ T cells were carried out on CD161^+^CD8^+^ T cell subsets.

### Circulating CD161^+^CD8^+^ T cells in rhesus macaques express tissue-homing markers with Th1/Th17 type phenotype

**Fig 2:**
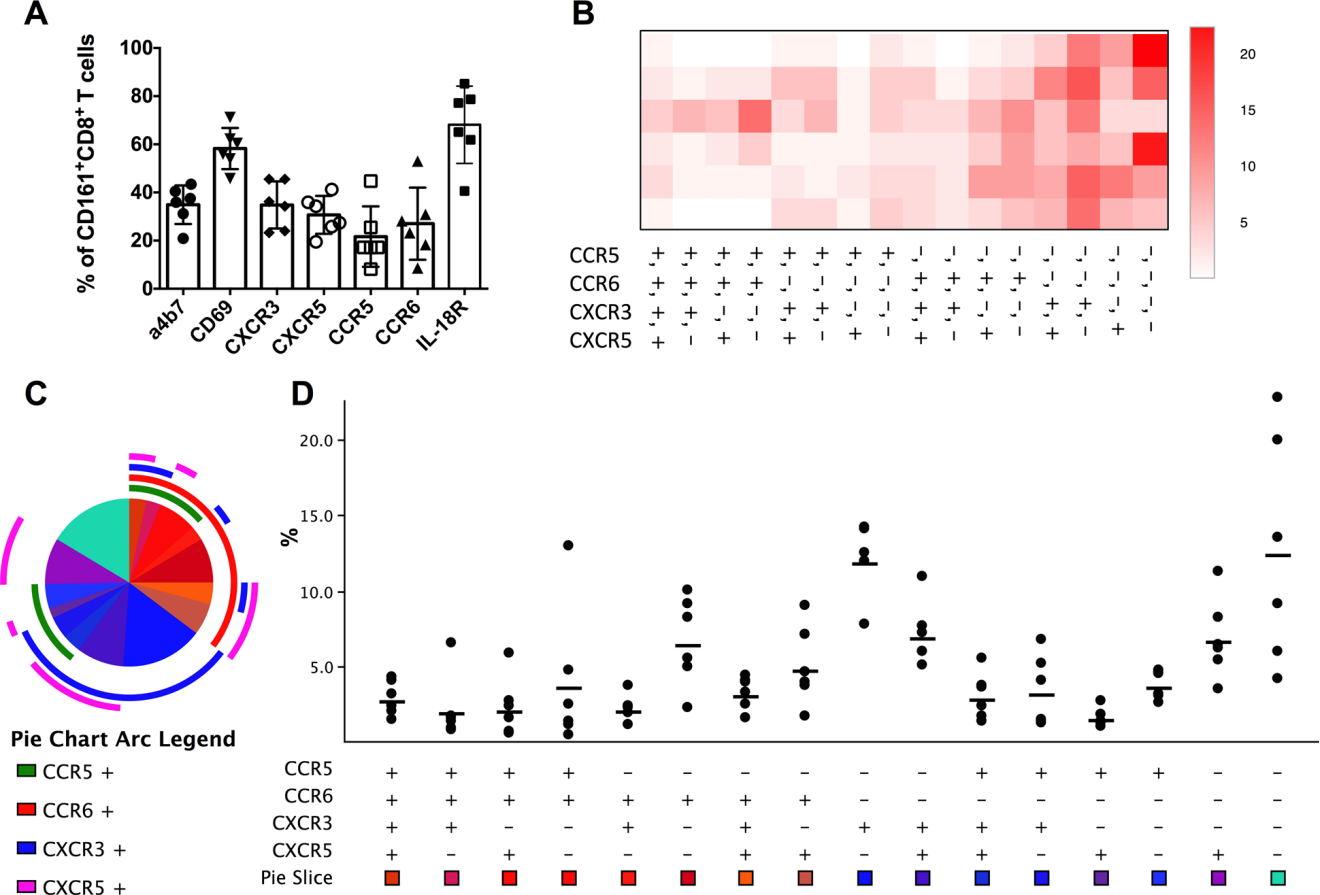
Trafficking marker expression profile of peripheral blood CD161^+^ CD8^+^ T cells in rhesus macaques. (A) Frequencies of expression of the trafficking markers α4β7, CD69, CXCR3, CXCR5, CCR5, CCR6, and IL-18Rα by *ex vivo* CD161^+^CD8^+^ T cells in the circulation of healthy rhesus macaques (Mean and SEM shown for data obtained from six animals). (B) Heat map showing relative expression of the chemokine receptors CCR5, CCR6, CXCR3, and CXCR5 generated using Prism 6 software. (C) Pie charts and arcs showing the proportion of different combinations of the chemokine receptors on CD161^+^CD8^+^ T cells as analyzed by Pestle and SPICE 5.3. (D) Boolean analysis showing the range and mean frequencies of CCR5, CCR6, CXCR3, and CXCR5 by CD161^+^CD8^+^ T cells in the 6 rhesus macaques.

Tissue-resident CD161^+^CD8^+^ T cells have been reported in the liver and intestine of humans [6,8,15,22]. Therefore, the surface marker expression pattern of circulating macaque CD161^+^CD8^+^ T cells was evaluated by flow cytometry in freshly isolated PBMC from healthy rhesus macaques utilizing the trafficking markers α4β7, CXCR3, CXCR5, CCR5, CCR6, IL-18Rα, and CD69. Surface expression levels of IL18Rα (mean = 68.1%, range: 40.6–85.2%), a receptor expressed on Th1 polarized cells in blood and tissues, and CD69 (mean = 58.2%, range: 45.8–71.2%), also a marker of activation and tissue residence, were most prominent among all the markers examined (Fig 2A). Additionally, 35% of CD161^+^CD8^+^ T cells expressed the gut homing marker α4β7 and displayed chemokine receptor expression pattern of Th1/Th17 cells with 35% and 22% expression of the Th1-associated CXCR3 and CCR5 molecules and 27% of the Th17-associated CCR6 molecule (Fig 2A). Circulating CD161^+^CD8^+^ T cells also expressed 31% of CXCR5, the marker associated with T cell trafficking in lymphoid tissue to B cell zone/follicles, suggesting homing to mucosal associated lymphoid tissue (MALT).

Analysis of the combinatorial expression of the chemokine receptors on ex vivo circulating CD161^+^CD8^+^ T cells by utilizing Spice software revealed a significant proportion of only CXCR3 expressing cells suggesting a Th1 type functionality of these cells (Fig 2A–2B). Furthermore, analysis of relative expression of the chemokine receptors CXCR3, CXCR5, CCR5, and CCR6 as a heat map showed CCR6 as the most common marker expressed with CXCR5 or CXCR3 (Fig 2B–2C), implying a Th1/Th17 profile in CD161^+^CD8^+^ T cells. The other two noticeable expression patterns in the heat map were CCR6 alone and CXCR3/CXCR5 co-expressing phenotype (Fig 2B–2D). Overall, circulating CD161^+^CD8^+^ T cells in rhesus macaques displayed a chemokine receptor expression phenotype consistent with homing to inflammatory sites and tissues.

### Th1/Th17 polyfunctional potential of circulating CD161^+^CD8^+^ T cells in rhesus macaques

**Fig 3:**
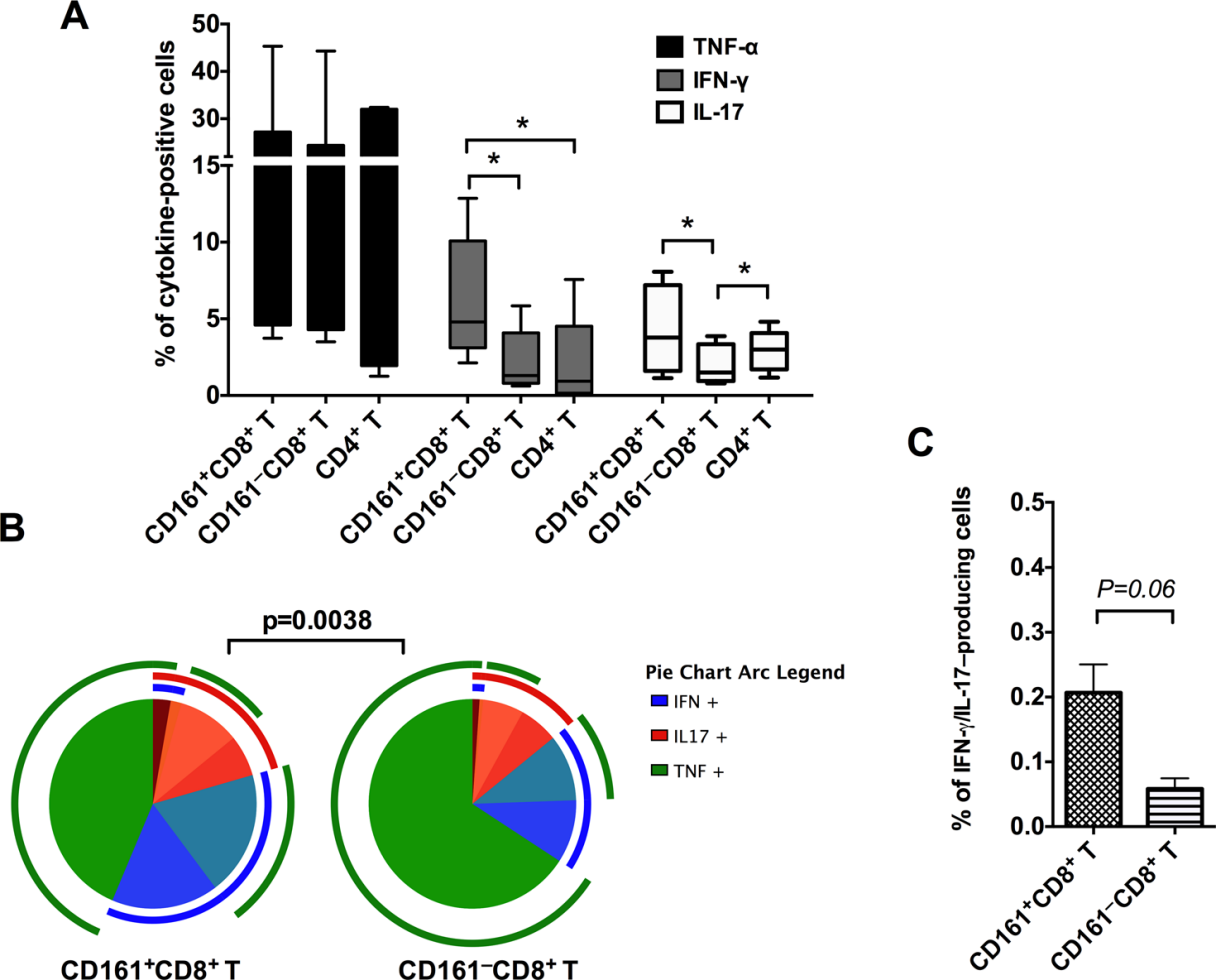
Cytokine production by circulating CD161^+^ CD8^+^ T cells in rhesus macaques. (A) Frequency of cytokine-secreting CD161^+^CD8^+^ T cells, CD161^–^CD8^+^ T cells, and CD4^+^ T cells in mitogen-stimulated PBMC of uninfected rhesus macaques (n = 6–7). Intracellular cytokine staining was performed on freshly isolated cells stimulated for 16h with PMA/Ionomycin. Data shows mean and SEM after subtraction of background in unstimulated cells incubated with medium alone. (B) Pie charts and arcs showing the expression of different combinations of the cytokines IFN-γ, TNF-α, and IL-17 by CD161^+^CD8^+^ T cells as analyzed by Pestle and SPICE 5.3. (C) Frequency of IFN-γ^+^IL-17^+^ co-producing cells in CD161^+^CD8^+^ T cell and CD161^–^CD8^+^ T cell subsets respectively. Asterisks denote significant difference (p = 0.03) calculated by the Wilcoxon matched-pairs signed rank test.

To further explore the effector functions of macaque CD161^+^CD8^+^ T cells, freshly isolated PBMC were stimulated with mitogen (PMA/Ca-Ionomycin) for 12 hours and intracellular production of the Th1 cytokines TNF-α and IFN-γ, and the Th17 cytokine IL-17 were analyzed. TNF-α dominated the cytokine response in CD161^+^CD8^+^ T cells (mean = 18%, range = 3.5–45%) and was similar to that in CD161^–^CD8^+^ T cell (mean = 16.2%) and CD4^+^ T cell subsets (mean = 16.4%; Fig 3A). However, IFN-γ production was the highest in CD161^+^CD8^+^ T cells and differed significantly from both CD4^+^ T cells and CD161^–^CD8^+^ T cells (*p* = 0.03; Fig 3A). Additionally, CD161^+^CD8^+^ T cells produced significantly higher levels of IL-17 than CD161^+^CD8^+^ T cells (*p* = 0.0156; Fig 3A). No significant differences were observed in IL-17 production between CD161^+^CD8^+^ T cells and CD4^+^ T cells. Thus, the CD161^+^CD8^+^ T cells cytokine function confirmed the Th1/Th17 phenotype observed in the surface marker expression analysis (Fig 2). The polyfunctionality of CD161^+^CD8^+^ T cells was analyzed by evaluating the simultaneous production of IFN-γ/TNF-α/IL-17 utilizing Spice software. In comparison to CD8^+^ T cells that do not express CD161, the CD161^+^CD8^+^ T cells had a significantly different combinatorial cytokine response with higher IFN-γ^+^IL-17^+^ co-producing ability (Fig 3B–3C).

### CD161^+^CD8^+^ T cells are enriched in mucosal tissues and largely comprised of γδ T cells and TCR Vα7.2^+^ MAIT cells

**Fig 4:**
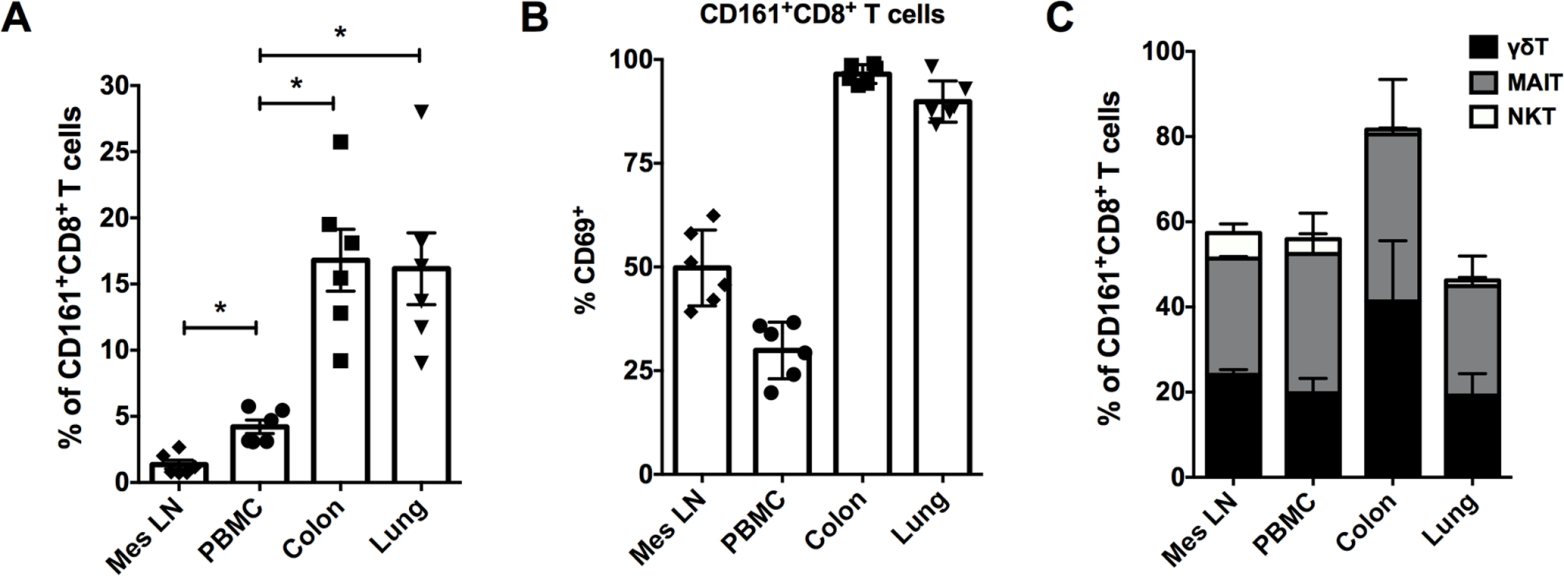
Frequency and composition of CD161^+^ CD8^+^ T cells in tissues. (A) CD161^+^CD8^+^ T cell frequencies in matched samples from PBMC, mesenteric lymph nodes (mes LN), colon and lung tissue of rhesus macaques (n = 6). (B) CD69 expression on CD161^+^CD8^+^ T cells in PBMC, mes LN, colon and lung. (C) Frequencies of MAIT cells (CD161^+^ Vα7.2^+^ T cells), γδ T cells (pan TCR γδ^+^), and iNKT (PBS-57-loaded CD1d TM^+^) cells respectively in CD161^+^CD8^+^ T cells of un-infected rhesus macaques. Asterisks denote significant differences (p = 0.03) calculated by the Wilcoxon matched-pairs signed rank test.

The results from phenotypic characterization of circulating CD161^+^CD8^+^ T cells also showed expression of markers of tissue-trafficking/residence (α4β7 and CD69, Fig 2A). Moreover, CD161^+^ T cells are reportedly enriched in the liver, colon, epithelium, and duodenum of humans [6,8,14,15,22]. Therefore, the frequencies of CD161^+^CD8^+^ T cells were analyzed in mesenteric lymph nodes and mucosal tissues (colon and lungs) of rhesus macaques. The CD161^+^CD8^+^ T cells were found to be enriched in both the mucosal tissue sites of rhesus macaques. Colon displayed a mean frequency of 16.8% (range = 9.2–25.7%), and lungs contained 16.3% (range = 9–28%) of total CD8^+^ T cells respectively, which was significantly higher than matched peripheral blood cells (Fig 4A). Interestingly, CD161^+^CD8^+^ T cells were rare in mesenteric lymph nodes, being significantly lower than even in peripheral blood (Fig 4A). Similar to PBMC, the CD161^+^ T cells in the mucosal tissues were predominantly CD8^+^ subsets, whereas in mesenteric lymph nodes they comprised equal proportions of CD8^+^, CD4^+^ and CD4^–^CD8^–^ cell subsets (S3 Fig). CD69 has been described as a marker of tissue retention and of tissue-resident memory T cell populations [23,24]. CD69 expression was therefore examined on T cells from different tissues, and was found to be almost entirely expressed by mucosal CD161^+^CD8^+^ T cell subsets (mean = 96.5% in colon and 90% in lung; Fig 4B). Despite being a rare subset in mesenteric lymph nodes, the CD161^+^CD8^+^ T cells had average 50% CD69 expression in comparison to 30% in PBMC (Fig 4B), likely indicative of their tissue-resident phenotype but lower level of activation in lymphoid tissue in comparison to mucosal tissue compartments.

Since high levels of CD161 expression are associated with innate T cell populations (including MAIT cells, γδ T cells, and iNKT cells) in humans [11], the composition of CD161^+^CD8^+^ T cells in blood and tissues was evaluated by flow cytometry using surface markers for specific subsets of innate T cells. Antibodies for Vα7.2 T cell receptor (TCR), γδ TCR, and antigen-loaded CD1d tetramers were utilized to examine frequencies of MAIT cells, γδ T cells, and iNKT cells respectively in CD161^+^CD8^+^ T cells of un-infected rhesus macaques. Taken together, these three subsets of innate T cells accounted for 56% of circulating CD161^+^CD8^+^ T cells, with majority (up to 95%) of them comprising of MAIT cells and γδ T cells (Fig 4C). However, only 3.5% of the total CD161^+^CD8^+^ T cells comprised of iNKT cells (Fig 4C). Similar to peripheral blood, CD161^+^CD8^+^ T cells in mesenteric lymph nodes, lungs and colon also comprised mainly of MAIT cells and γδ T cells (46–82%) in roughly equal proportions (Fig 4C), suggesting that majority of CD161^+^CD8^+^ T cells in macaques also represent innate T cell subsets.

### Mucosal tissue derived CD161+CD8+ T cells produce higher IL-17 than peripheral blood cells

**Fig 5:**
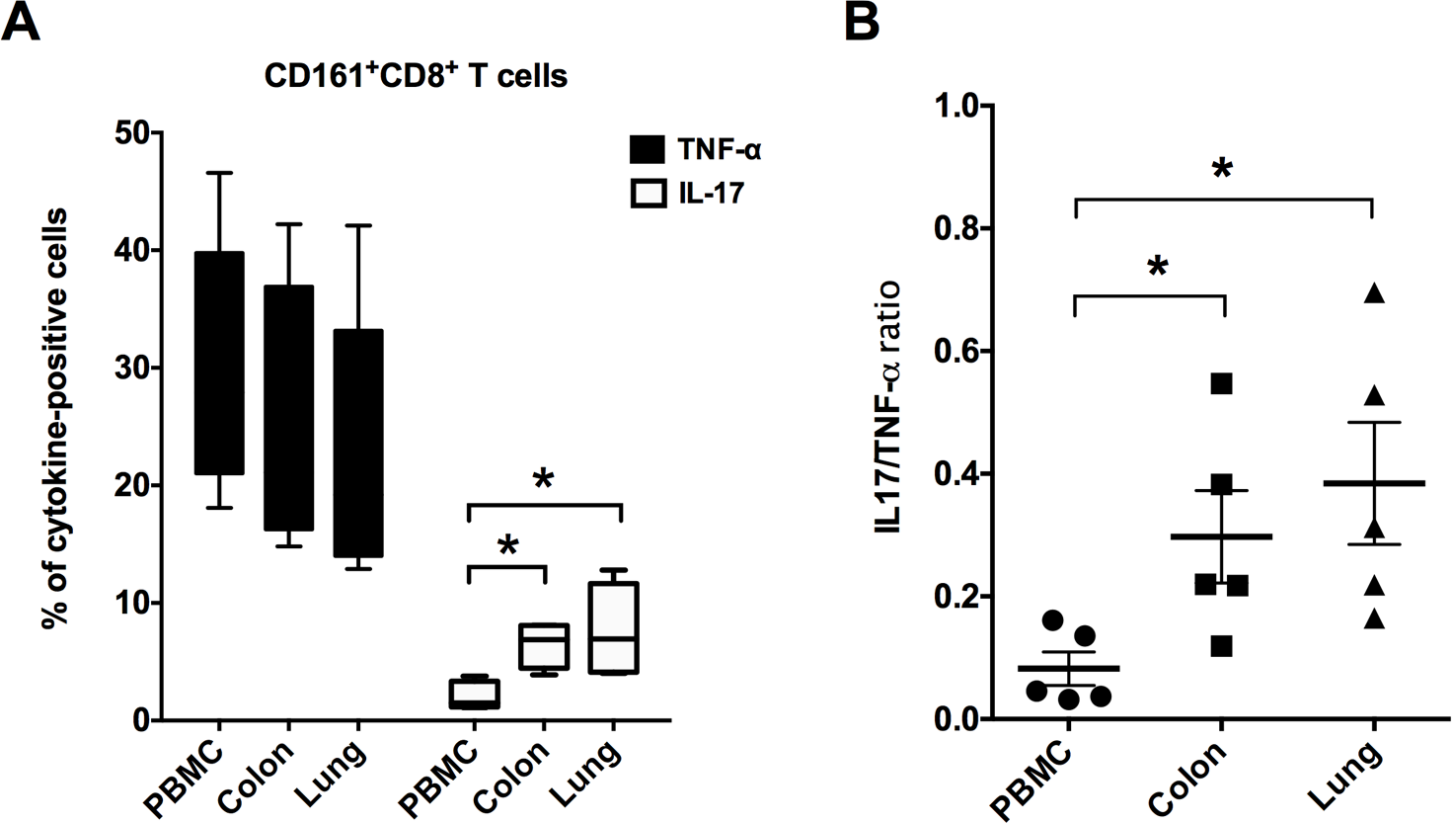
CD161^+^ CD8^+^ T cell cytokine production in mucosal tissues. (A) Intracellular levels of TNF-α and IL-17 cytokines by mitogen-stimulated CD161^+^CD8^+^ T cells in PBMC, colon and lung of uninfected rhesus macaques (n = 5–6). Intracellular cytokine staining was performed on freshly isolated cells stimulated for 16h with PMA/Ionomycin. Data shows mean and SEM after subtraction of background in unstimulated cells incubated with medium alone. (B) Ratios of IL-17 to TNF-α in CD161^+^CD8^+^ T cells isolated from PBMC, colon and lung showing increase in Th17-type responses in mucosal tissues. Asterisks denote significant differences (p<0.05) calculated by the Wilcoxon matched-pairs signed rank test.

The cytokine-effector functions of mucosal CD161^+^CD8^+^ T cells were analyzed similar to earlier described in PBMC, following stimulation with PMA/Ca-Ionomycin for 12 hours. Intracellular production of the Th1 cytokine TNF-α, and the Th17 cytokine IL-17 were analyzed and compared between PBMC and mucosal tissues. Similar to that observed in PBMC, CD161^+^CD8^+^ T cells in mucosal tissues produced high levels of TNF-α (mean = 23–26%, range = 13–42%; Fig 5A). On the other hand, mucosal CD161^+^CD8^+^ T cells demonstrated significantly higher intracellular levels of IL-17 than peripheral blood CD161^+^CD8^+^ T cells (Fig 5A). Thus, the ratios of IL17 to TNF-α positive CD161^+^CD8^+^ T cells were observed to be significantly higher (p = 0.03) in lungs and colon in comparison to PBMC (Fig 5B), suggesting an enhanced Th17 function in mucosal sites.

**Fig 6:**
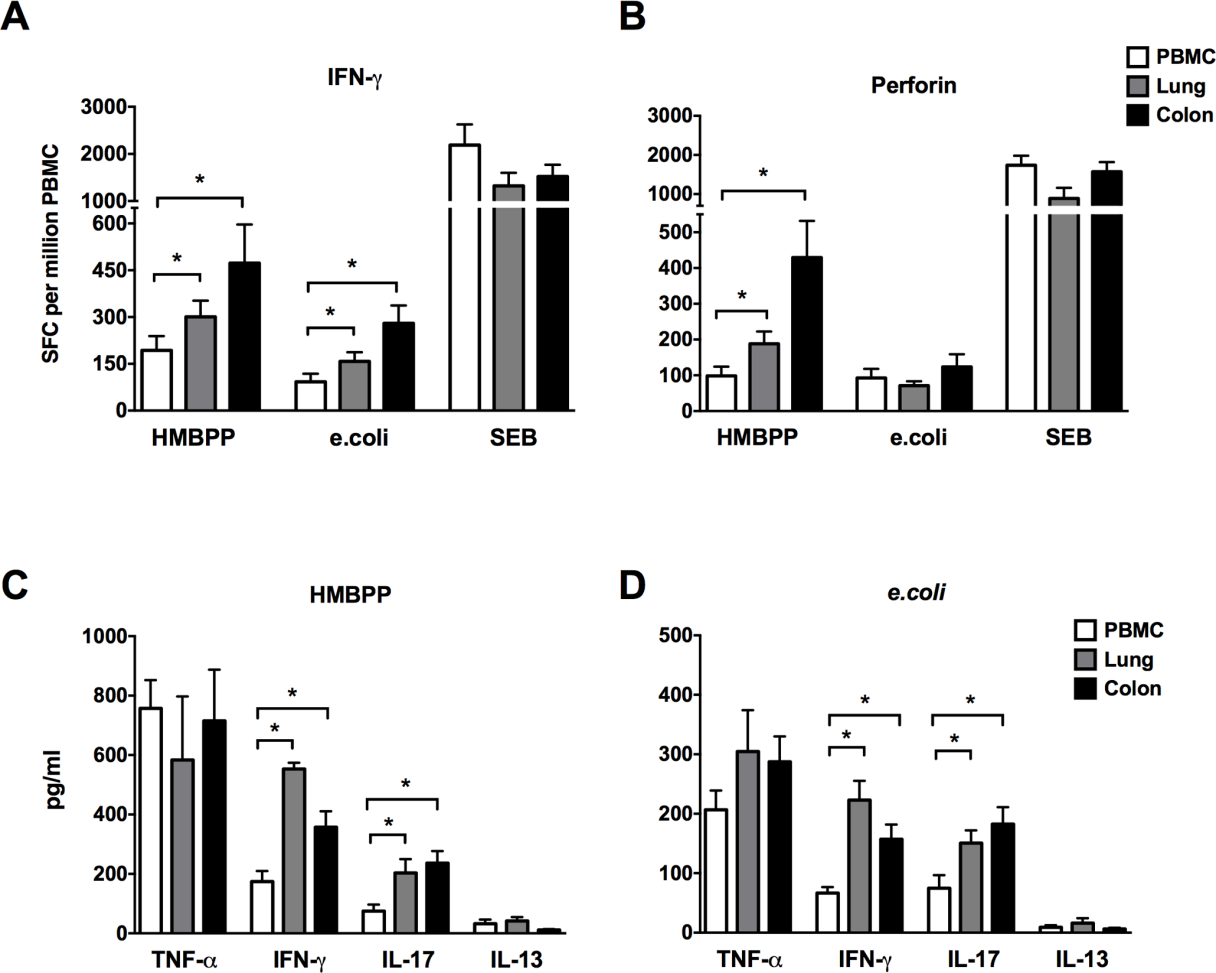
Innate CD161^+^ T cell cytokine secretion and cytotoxic response in blood and mucosal tissues. HMBPP-specific and fixed *E*.*coli*-induced secretion of IFN-γ (A) and Perforin (B) was analyzed in lymhocytes from PBMC, lung and colonic mucosa using enzyme-linked immunosorbent spot (ELISpot) assay. Enzyme-linked immunosorbent assay (ELISA) for TNF-α, IFN-γ, IL-17 and IL-13 in culture supernatants collected 16 hours following HMBPP (C) and *E*.*coli* (D) stimulation of lymhocytes from PBMC, lung and colonic mucosa. Data obtained from 6 rhesus macaques. Asterisks denote significant differences (p<0.05) calculated by the Wilcoxon matched-pairs signed rank test.

Since γδ T cells and MAIT cells accounted for the majority of CD161^+^CD8^+^ T cells (Fig 4C), the cytokine and cytotoxic effector function of these subsets was further examined following stimulation by their agonists. A dominant subset of γδ T cells is comprised of Vγ2Vδ2 T cells, which exist only in human and nonhuman primates [25,26] and recognize the bacterial phosphoantigen (E)-4-hydroxy-3-methyl-but-enyl pyrophosphate (HMBPP). MAIT cells, on the other hand, recognize ligands bound to MR1 originating from metabolic intermediates of bacterial riboflavin synthesis pathway [27], and thus, PFA fixed *e*.*coli* has been used to stimulate MAIT cells [19]. Therefore, in this study HMBPP and paraformaldehyde-fixed *e*.*coli* were used to stimulate γδ T cells and MAIT cells respectively. Following *ex vivo* stimulation with HMBPP, greater γδ T cell cytokine and cytotoxic effector responses were observed in lungs and colon compared to PBMC as evidenced by significantly higher IFN-γ and perforin spot forming cells (Fig 6A–6B), in concordance with the increased frequencies of the parent CD161^+^CD8^+^ T cell population observed in mucosal tissues (Fig 4A). Similarly, significantly higher IFN-γ spot forming cells were observed in *e*.*coli*-stimulated MAIT cells in lymphocytes isolated from lungs and colon (Fig 6A), but there was no enhanced cytotoxicity (perforin spot forming cells) of mucosal tissue-derived cells with respect to PBMC (Fig 6B). The cytokine secretion by unfractionated γδ T cells and MAIT cells was further evaluated for an extended panel of Th1 (TNF-α and IFN-γ), Th17 (IL-17) and Th2 (IL-13) cytokines by ELISA. TNF-α was the dominant Th1 cytokine secreted in response to γδ T and MAIT cell stimulation with HMBPP and paraformaldehyde-fixed *e*.*coli*, and did not differ between peripheral blood and mucosal tissue compartments (Fig 6C). However, in concordance with ELISPOT data, IFN-γ secretion was significantly higher by stimulated γδ T and MAIT cells present in lymphocytes isolated from lungs and colon in comparison to PBMC (Fig 6C). Also, as earlier observed in ICS of mitogen stimulated CD161^+^CD8^+^ T cells (Fig 5A), IL-17 secretion was significantly higher in lungs and colon following HMBPP and *e*.*coli* stimulation (Fig 6C–6D). IL-13 secretion was barely detectable in all of the tissues (Fig 6C–6D), suggesting paucity of Th2 cytokine secretion in HMBPP and *e*.*coli* stimulated cells. Thus, CD161^+^CD8^+^ T cells are enriched in mucosal tissues of rhesus macaques and comprise mainly of γδ T and MAIT cells that display enhanced IFN-γ and IL-17 cytokine secreting ability. Additionally, mucosal γδ T cells demonstrated greater perforin-secreting cytotoxic potential than that of peripheral blood (Fig 6B). Taken together these data suggest that CD161^+^ innate T cell subsets have greater cytokine and cytotoxic effector functions in tissue sites of constant microbial exposure.

## Discussion

It is becoming increasingly clear that innate T cells have an important role in immune responses to invading pathogens. Human CD161-expressing T cells have a characteristic transcriptional signature associated with innate-like function, display antiviral potential, and are enriched in tissues [7,14], suggesting that their effector functions are significant in tissue specific immune responses. The current study evaluated the phenotypic and functional characteristics of CD161-expressing T cells in peripheral blood and tissues of the nonhuman primate model of rhesus macaques. The results demonstrate that, like their human counterparts, rhesus macaque CD161^+^ T cells predominantly express CD8, display Th1/Th17 type functionality and are enriched in mucosal tissues.

Circulating CD161+ T cells in rhesus macaques displayed similar range and mean frequency to that reported in healthy humans [7,28]. Although, there was a lack of clear phenotypic distinction into CD161^++^, CD161^+^ and CD161^low^ cells, which is characteristic of human MAIT cells, other innate T cells including iNKT and γδ T cells, and conventional T cells respectively [6,11,21]. The presence of a similar trend for CD161 MFI on these distinct T cell subsets, however, suggests that macaque CD161^+^ T cells are analogous to human CD161-expressing T cells and comprise of the diverse group of innate T cells including MAIT, iNKT and γδ T cells, along with unknown subsets. Moreover, the mucosal trafficking marker and chemokine/cytokine receptor expression pattern of circulating CD161^+^CD8^+^ T cells, the major subset of CD161^+^ T cells, indicate that they are an important component of the mucosal tissue-homing T cell populations with Th1 and Th17 type potential. IL-18R was the most prominent cytokine receptor expressed on circulating CD161^+^ CD8^+^ T cells. Since IL-18R is reported to be one of the signature genes in human MAIT and Tc17 cells that emerge early after birth and appear to be pre-programmed towards type-17 immune responses [29], high expression of IL-18R by macaque CD161^+^CD8^+^ T cells further confirms their functional equivalence to human CD161^+^CD8^+^ T cells. Additionally, the CD161^+^CD8^+^ T cells in peripheral blood of rhesus macaques expressed CD69, α4β7, and CXCR5. Circulating lymphocytes associated with homing to the gastrointestinal tract are known to express the MAdCAM-1 ligand α4β7 integrin [30]. Besides, tissue-infiltrating lymphocytes are characterized by an “activated” phenotype with greater expression of CD69 [31]. Also, CXCR5 expression, which is a hallmark of follicular helper T cells, enables homing in response to CXCL13 produced within B cell follicles [32]. Thus, expression of CD69, α4β7, and CXCR5 by majority of macaque CD161^+^CD8^+^ T cells indicates their homing to gut and gut-associated lymphoid systems. Migration of T cells from blood through the expression of specific chemokine and adhesion molecules is important for tissue-specific immune functions. It is established that chemokine receptors CCR5, and CXCR3 are highly associated with Th1 type “tissue-resident” T cells found in extralymphoid tissues [31], and CCR6 expression characterizes Th17 cells [33]. Therefore, expression of CXCR3, CCR5 and CCR6, by a quarter to a third of circulating CD161^+^CD8^+^ T cells suggests presence of subsets with Th1 or Th17 type function.

Based on the analysis of cytokine effector functions in peripheral blood CD161^+^CD8^+^ T cells, these cells were found to produce TNF-α, IFN-γ and IL-17, but not the Th-2 cytokine IL-13 following *in vitro* stimulation. Furthermore, greater IFN-γ and IL-17 production by CD161-expressing CD8^+^ T cells than CD161^–^ cells indicate an association of CD161-expression with enhanced Th1/Th17 functions in CD8^+^ T cells. These observations are consistent with similar studies on CD161^+^ T cells in human blood [6,7,28,34]. The enrichment of CD8^+^ subsets in CD161^+^ T cells is interesting, and is likely a part of the transcriptional signature/program that allows for their phenotypic similarity to NK cells and innate-like functions [7].

Recent studies have indicated increased frequencies of Tc17-like CD161^+^ T cells in human extralymphoid tissues including gut, lungs, liver and skin [6,8,15,22,28], suggesting an important role in local immune responses of diverse tissues. A similar enrichment of CD161^+^ T cells in the colon and lungs, but not in mesenteric lymph nodes, of uninfected rhesus macaques was observed An important finding of this study is the presence of significant proportions of γδ T cells and MAIT cells in the larger population of CD161^+^ T cells in peripheral blood as well as lymphoid and mucosal tissues, suggesting that CD161 expression could also serve as a surrogate marker to distinguish the overall innate T cell subsets from the conventional T cells. Besides greater IFN-γ, and IL-17 responses to γδ T and MAIT cell stimulants (HMBPP and fixed *E*.*coli* respectively), stimulation of the γδ subsets of CD161^+^ T cells also demonstrated higher perforin release in the mucosal tissues, indicating enhancement of cytotoxic potential. Fergusson *et al*., have recently demonstrated that EBV-, CMV- and influenza-specific antiviral CD8^+^ T cells contained a subset of CD161^+^ T cells that had significantly higher expression of Granzyme B and Perforin than the virus-specific CD161^–^ T cell subsets [14], indicating their cytotoxicity against virally-infected cells and a role in anti-viral immunity. It is likely that the larger group of CD161^+^ T cells comprises of a heterogeneous population of both peptide and non-peptide antigen-specific immune subsets that are pre-conditioned towards a Th1/Th17 type effector function. Interestingly, greater IL-17 and IFN-γ function of CD161^+^ T cells in the mucosal tissues of rhesus macaques suggest that this subset of T cells possibly develop effector functions through stimulation by their cognate antigens expressed by commensal microbes/endogenous ligands in mucosal tissues. Furthermore, IL-17 mediated immune responses play a crucial role in mucosal defense against microbial pathogens. CD4^+^ Th17 cells and innate immune cells including innate T cells and innale lymphoid cells (ILCs) contribute to IL-17 released during an inflammatory response [35]. These innate sources have been indicated to play a central role in the initiation of IL-17-dependent immune responses, in the interval during which conventional CD4+T cells respond to cognate antigens and initiate the Th17 cell differentiation program [35].

Overall, this study characterized the CD161^+^ subset of T cells in macaques and demonstrated that they are enriched in lungs and colon and possess Th1/Th17 cytokine producing and cytotoxic potential. These data suggest that the tissue-resident properties of CD161^+^CD8^+^ T cells along with their immune effector functions may have an important role in mucosal tissue-specific immunity. The results of this study corroborate the findings of several studies in humans that underscore the important Th1 and Th17 functions of CD161^+^ T cells in tissues [6,8,14,15,22,28]. Since longitudinal assessment and deep tissue access is required to address their role in mucosal diseases, it is essential to define their functions in the nonhuman primates as the pre-clinical model of important human diseases like HIV-AIDS and TB. Further studies are underway to investigate their distribution and functions in rhesus macaques infected with simian immunodeficiency virus (SIV) and *Mycobacterium tuberculosis* (Mtb) to understand the functions of CD161^+^CD8^+^ T cells in protection or pathogenesis of viral and bacterial infections. Defining CD161^+^ T cell functions in early immune responses to pathogens, particularly in tissues, may provide important information on their role in various human infectious diseases and thus aid the development of novel intervention strategies.

## Supporting Information

S1 Fig CD161 expression by innate T cell subsets in peripheral blood of rhesus macaques. (A) Overlay of histogram plots on one macaque PBMC sample showing expression of CD161 by CD3^+^ T cells, γδ TCR^+^ cells, PBS-57 loaded CD1d Tetramer^+^ iNKT cells, and TCR Vα7.2^+^ MAIT cells. (B). CD161 MFI (Median Fluorescence Intensity) of total T cells, γδ T cells, iNKT cells, and MAIT cells in PBMC from 5 healthy rhesus macaques. Asterisk denotes significant difference (p<0.05) calculated by the Wilcoxon matched-pairs signed rank test.
(TIFF)

S2 Fig Memory phenotype of circulating CD161^+^CD8^+^ T cells in comparison to total CD3^+^ T cells. (A) Representative flow plot showing the memory phenotype of CD3^+^ T cells and CD161^+^CD8^+^ T cells in PBMC of one healthy rhesus macaque. Dot plots show *ex vivo* staining of CD95 vs CD28 on the cells. (B). Scatter plot with bar showing mean percentage of CD95 expressing cells in total T cells and CD161^+^CD8^+^ T cells in PBMC from 9 healthy rhesus macaques. Error bar denotes SEM. Asterisks denote significant difference (** p<0.01) calculated by the Wilcoxon matched-pairs signed rank test.
(TIFF)

S3 Fig Expression of CD4 and CD8 by CD161^+^CD8^+^ T cells in different tissue compartments of rhesus macaques. Stacked bars showing mean values and SEM for T cell subset distribution based on expression of CD4 and CD8 co-receptors by CD161^+^ T cells in peripheral blood, colon, lung and mesenteric lymph node lymphocytes obtained from necropsy tissues of 4 rhesus macaques.
(TIFF)
