# Supplementary figures and images for: Enhanced Th1/Th17 Functions of CD161+ CD8+ T Cells in Mucosal Tissues of Rhesus Macaques
Source: PLoS One. 2016 Jun 16;11(6):e0157407. doi: 10.1371/journal.pone.0157407 (PMC4911052; doi:10.1371/journal.pone.0157407)

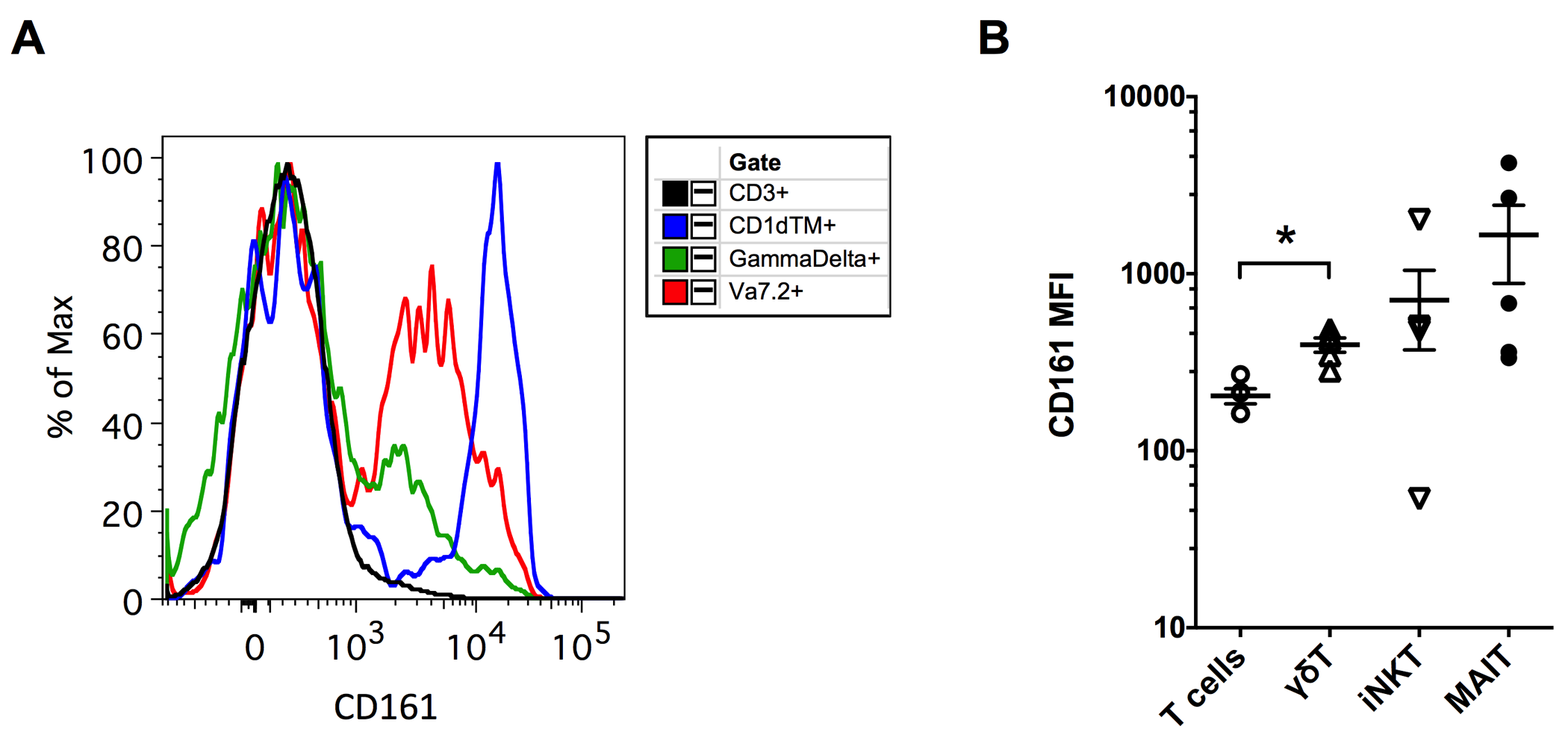

Supplement: S1 Fig — (A) Overlay of histogram plots on one macaque PBMC sample showing expression of CD161 by CD3+ T cells, γδ TCR+ cells, PBS-57 loaded CD1d Tetramer+ iNKT cells, and TCR Vα7.2+ MAIT cells. (B). CD161 MFI (Median Fluorescence Intensity) of total T cells, γδ T cells, iNKT cells, and MAIT cells in PBMC from 5 healthy rhesus macaques. Asterisk denotes significant difference (p<0.05) calculated by the Wilcoxon matched-pairs signed rank test. (TIFF) [file pone.0157407.s001.tiff]

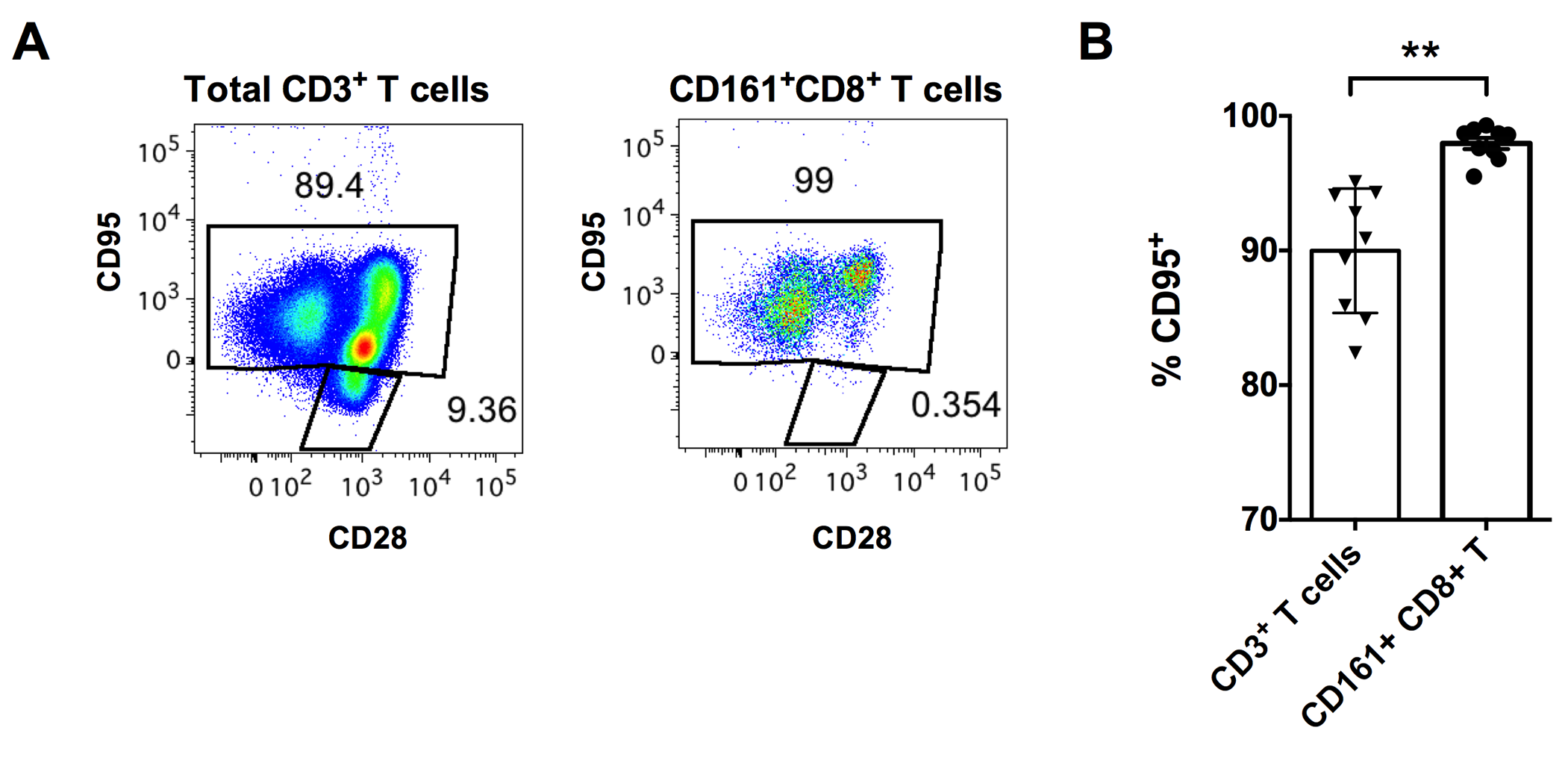

Supplement: S2 Fig — (A) Representative flow plot showing the memory phenotype of CD3+ T cells and CD161+CD8+ T cells in PBMC of one healthy rhesus macaque. Dot plots show ex vivo staining of CD95 vs CD28 on the cells. (B). Scatter plot with bar showing mean percentage of CD95 expressing cells in total T cells and CD161+CD8+ T cells in PBMC from 9 healthy rhesus macaques. Error bar denotes SEM. Asterisks denote significant difference (** p<0.01) calculated by the Wilcoxon matched-pairs signed rank test. (TIFF) [file pone.0157407.s002.tiff]

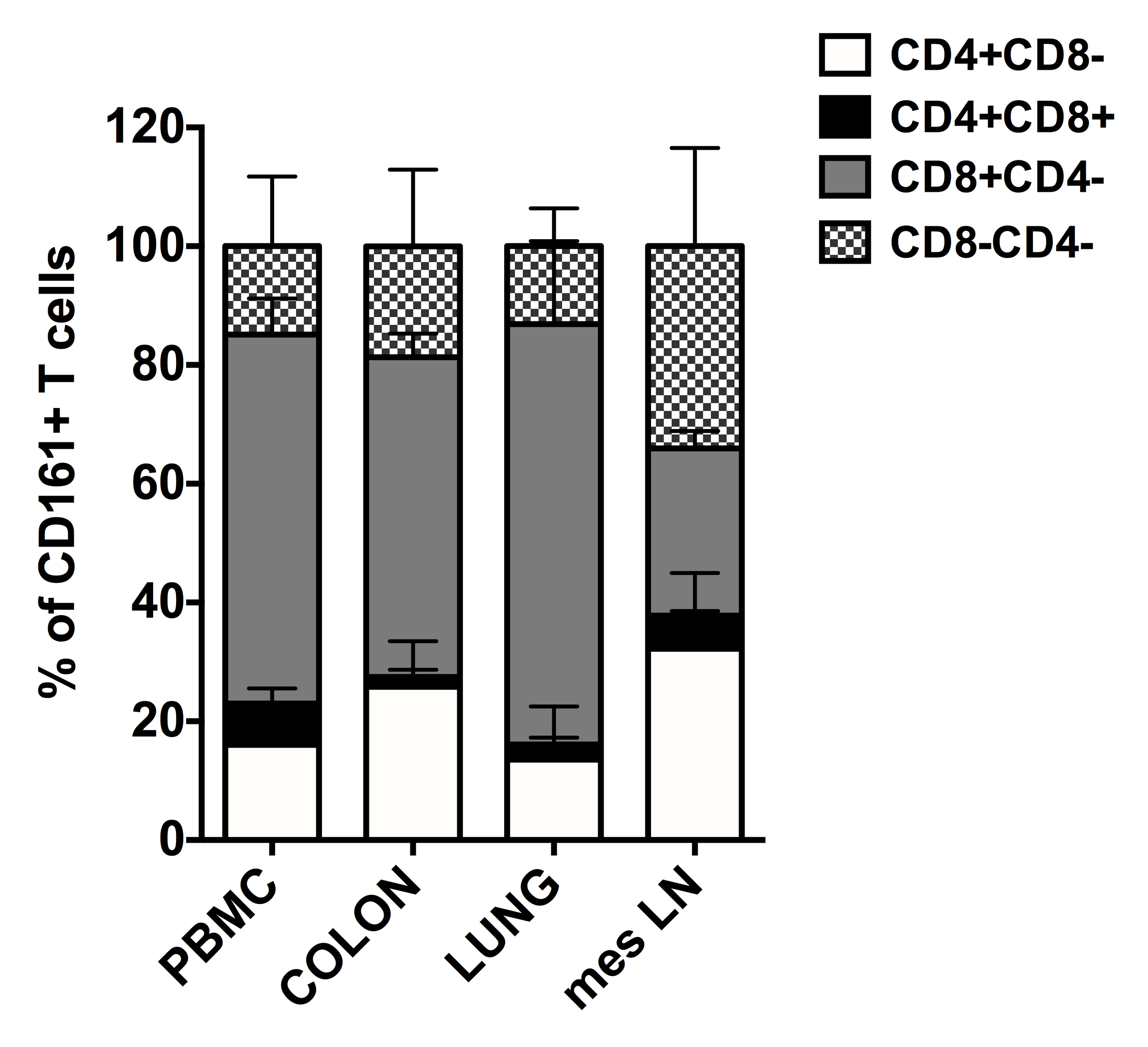

Supplement: S3 Fig — Stacked bars showing mean values and SEM for T cell subset distribution based on expression of CD4 and CD8 co-receptors by CD161+ T cells in peripheral blood, colon, lung and mesenteric lymph node lymphocytes obtained from necropsy tissues of 4 rhesus macaques. (TIFF) [file pone.0157407.s003.tiff]
